# The Modification of Plant Cell Wall Polysaccharides in Potato Plants during *Pectobacterium atrosepticum*-Caused Infection

**DOI:** 10.3390/plants10071407

**Published:** 2021-07-09

**Authors:** Vladimir Gorshkov, Ivan Tsers, Bakhtiyar Islamov, Marina Ageeva, Natalia Gogoleva, Polina Mikshina, Olga Parfirova, Olga Gogoleva, Olga Petrova, Tatyana Gorshkova, Yuri Gogolev

**Affiliations:** 1 Kazan Institute of Biochemistry and Biophysics, Federal Research Center Kazan Scientific Center of Russian Academy of Sciences, 420111 Kazan, Russia; b.islamov@knc.ru (B.I.); ageeva@kibb.knc.ru (M.A.); gogoleva@kibb.knc.ru (N.G.); mikshina@kibb.knc.ru (P.M.); olga.parfirova@kibb.knc.ru (O.P.); olga.petrova@kibb.knc.ru (O.P.); gorshkova@kibb.knc.ru (T.G.); gogolev@kibb.knc.ru (Y.G.); 2 Laboratory of Plant Infectious Diseases, Federal Research Center Kazan Scientific Center of Russian Academy of Sciences, 420111 Kazan, Russia; i.Tsers@knc.ru (I.T.); gogolewaoa@yandex.ru (O.G.); 3 Institute of Fundamental Medicine and Biology, Kazan Federal University, 420111 Kazan, Russia

**Keywords:** plant soft rot, *Pectobacterium*, plant cell wall, pectic compounds, cross-linking glycans, virulence factors

## Abstract

Our study is the first to consider the changes in the entire set of matrix plant cell wall (PCW) polysaccharides in the course of a plant infectious disease. We compared the molecular weight distribution, monosaccharide content, and the epitope distribution of pectic compounds and cross-linking glycans in non-infected potato plants and plants infected with *Pectobacterium atrosepticum* at the initial and advanced stages of plant colonization by the pathogen. To predict the gene products involved in the modification of the PCW polysaccharide skeleton during the infection, the expression profiles of potato and *P. atrosepticum* PCW-related genes were analyzed by RNA-Seq along with phylogenetic analysis. The assemblage of *P. atrosepticum* biofilm-like structures—the bacterial emboli—and the accumulation of specific fragments of pectic compounds that prime the formation of these structures were demonstrated within potato plants (a natural host of *P. atrosepticum*). Collenchyma was shown to be the most “vulnerable” tissue to *P. atrosepticum* among the potato stem tissues. The infection caused by the representative of the Soft Rot *Pectobacteriaceae* was shown to affect not only pectic compounds but also cross-linking glycans; the content of the latter was increased in the infected plants compared to the non-infected ones.

## 1. Introduction

The plant cell wall (PCW) is considered to be a battleground of plant–pathogen interactions. As a part of the defense strategy, the PCW is widely shown to be strengthened by lignification and suberinization as well as enriched by various antimicrobial compounds, such as PR-proteins, phytoalexins, etc. [1,2,3]. The major components of the PCW are polysaccharides: cellulose, cross-linking glycans, and pectins. The structure of the polysaccharide skeleton of the PCW also evidently undergoes a transformation during infectious diseases, since most (if not all) phytopathogens produce extracellular enzymes degrading PCW polysaccharides and/or proteins (e.g., expansins) that disturb the interaction between different polysaccharides. In addition, many phytopathogens were shown to exploit PCW enzymes and proteins of the host plant to cause PCW loosening mediated by the host itself [4,5,6]. It is known that the directed modification of the structure and properties of the particular PCW polysaccharides (e.g., by plant gene regulation/knockout) can influence plant resistance to pathogens [7,8,9]. However, to the best of our knowledge, no comprehensive studies on the modification of the whole PCW polysaccharide ensemble within the infected plant were performed for any infectious disease except for the investigation of pectin destruction in tobacco plants after infection with *Pectobacterium atrosepticum* (*Pba*) [10].

*Pba* belongs to a group of devastating soft rot-causing phytopathogenic bacteria (Soft Rot *Pectobacteriaceae*, SRP). These bacteria produce multiple PCW-degrading enzymes (PCWDEs), predominantly pectin-degrading, causing tissue maceration [11]. The plant polysaccharide degradation products are used by *Pba* as a growth substrate as well as “building blocks” for the assemblage of the specific “multicellular” biofilm-like structures—bacterial emboli [10,12]. These structures are formed only in the primary xylem vessels of the infected plants. The primary extracellular matrix of bacterial emboli includes high molecular weight fragments of one of the pectic compounds, rhamnogalacturonan I (RG-I), which is released from the PCW into the vessel lumen and provides the consolidation of *Pba* cells in a holistic structure [10]. The release of RG-I occurs even before the bacterial cells invade a vessel and can, thus, be attributed to the action of only plant, but not bacterial, enzymes/proteins. Plant RG-I lyases, XETs, and expansins encoded by plant genes upregulated during *Pba*-caused infection in tobacco were predicted to be involved in the release of RG-I [13].

The analysis of the infection-associated PCW modification enabled us to unravel crucial steps of the formation of novel infection structures (namely, to identify the components of bacterial emboli matrix) and to reveal the *Pba*-induced plant susceptible response related to the upregulation of certain plant PCW-related genes, whose products cause the reorganization of the PCW and the release of RG-I required for the pathogen to assemble bacterial emboli. However, these investigations were performed on tobacco, which is an experimental host for *Pba* widely used to study *Pectobacterium*-caused pathogenesis. Thus, the *Pba*-induced PCW polysaccharide modifications, including those related to the release of RG-I, have not been analyzed in *Pba* natural host (potato). The possibility of bacterial emboli formation in *Pba*-infected potato plants has also not been assessed previously.

Therefore, the aim of our study was to characterize the process of PCW polysaccharide modification in potato plants at different stages of plant colonization by *Pba*. We carried out the biochemical and immunolocalization analyses of PCW polysaccharides and assessed if the bacterial emboli were formed in *Pba*-infected potato plants. In addition, since the modification of PCW polysaccharides during infectious diseases can be implemented by gene products of both pathogen and host plant, we performed the analysis of expression of PCW-related genes of both potato and *Pba* using RNA-Seq.

## 2. Results

**Figure 1:**
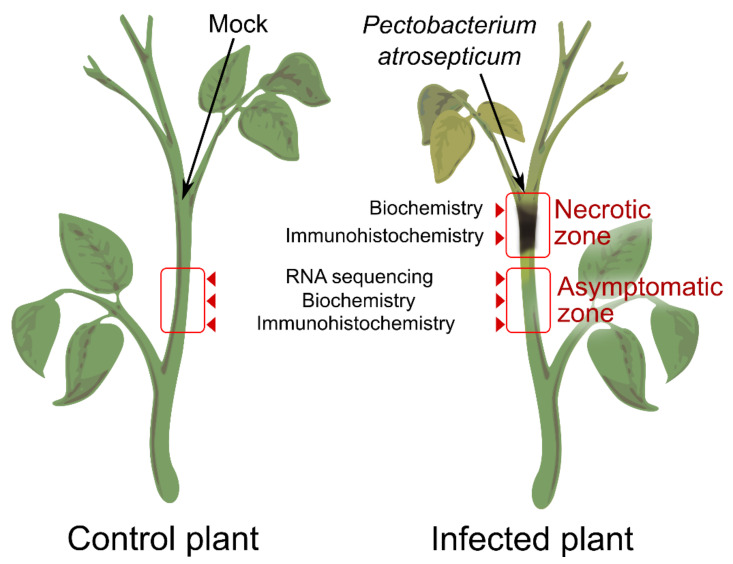
Scheme of the plant material (*Pectobacterium atrosepticum*-infected and control potato plants) collection for the plant cell wall-related gene expression analysis as well as biochemical and immunolocalization analyses of the plant cell wall polysaccharides.

To characterize the process of modification of PCW polysaccharides, two stem zones of the infected plants were analyzed: (1) the necrotic zone located around the inoculation point and (2) the zone located 1 cm below the necrotic zone (referred to as the asymptomatic zone) (Figure 1). The zone located 1 cm below the point of injection of sterile MgSO_4_ was taken from control non-infected plants. All three types of samples were analyzed using chromatography and immunocytochemistry; transcriptome profiling was performed only for asymptomatic zone and control plants, since in the necrotic zone, RNA was severely degraded.

### 2.1. Biochemical Analysis of PCW Polysaccharides during Pba-Caused Infection

Three fractions of PCW polysaccharides were analyzed: (1) the Na/K-phosphate buffer-extractable fraction that contained unbound or weakly bound PCW polysaccharides; (2) the ammonium oxalate-extractable fraction that predominantly included PCW-bound pectic compounds; and (3) potassium hydroxide (KOH)-extractable fraction that contained cross-linking glycans (CLGs) retained by cellulose. Fractions were analyzed by size-exclusion chromatography and the monosaccharide content was determined by ion chromatography.

#### 2.1.1. Polysaccharides of Buffer-Extractable Fraction

**Figure 2:**
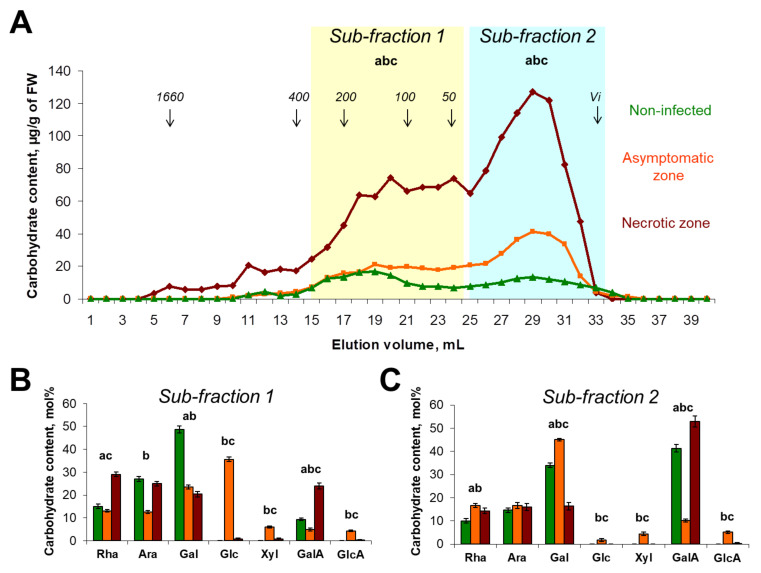
The molecular weight distribution and monosaccharide composition of plant cell wall polysaccharides extracted with 50 mM Na/K-phosphate buffer from the samples of stems of non-infected (green) and *Pectobacterium atrosepticum*-infected (orange and brown) potato plants: asymptomatic zone (orange) and necrotic zone (brown). (**A**) Elution profiles were obtained using size-exclusion chromatography on a Sepharose CL-4B column; pullulans were used as molecular weight markers; Vi—inclusion volume; values are the means (per g of fresh weight, FW) of three biological replicates. The monosaccharide composition was analyzed in different sub-fractions, i.e., (**B**) sub-fraction 1 and (**C**) sub-fraction 2, which are marked on elution profiles following hydrolyzation with trifluoride acetic acid; values are the means ±SD (mol%) of three biological replicates. Small letters a, b, and c point to the significant difference in the carbohydrate content within a particular sub-fraction (panel A) or in mol% content of a particular monosaccharide (panels B, C) (Mann–Whitney two-sided test, *p* < 0.05): a—control vs. necrotic zone; b—control vs. asymptomatic zone; c—asymptomatic zone vs. necrotic zone. Monosaccharide names are abbreviated as follows: Rha—rhamnose, Ara—arabinose, Gal—galactose, Glc—glucose, Xyl—xylose, GalA—galacturonic acid, GlcA—glucuronic acid.

The total content of carbohydrates in the buffer-extractable fraction was 2- and 6-fold higher in the asymptomatic and necrotic zones, respectively, compared to control plants. For each of three sample types, two main sub-fractions were revealed in the buffer-extractable fraction (Figure 2A). The first sub-fraction contained polymers eluting in the region from 400 kDa to 50 kDa (according to column calibration with pullulans), and carbohydrates of < 50 kDa represented the second sub-fraction. According to monosaccharide content, the first sub-fraction (higher molecular weight) of the control plants was predominantly composed of RG-I fragments (the ratio Rha/GalA ≈ 1) with Gal and Ara side chains, while the second sub-fraction (lower molecular weight) contained more polygalacturonic acid (PGA), since the ratio Rha/GalA was less than 0.25 (Figure 2B,C).

In the asymptomatic zone, the first sub-fraction included a high level of Glc (36 mol%) that presumably emerged due to starch hydrolysis (Figure 2B). Both the first and the second sub-fractions of the buffer-extractable fraction of the asymptomatic zone contained Rha (13 and 17 mol%), Ara (13 and 17 mol%), Gal (24 and 45 mol%), and GalA (5 and 10 mol%), which are the constituents of RG-I. In addition, 6 and 4 mol% of Xyl were revealed in these sub-fractions indicating that some CLGs (xylose-containing PCW polysaccharides xylan and/or xyloglucan) became unbound to the PCW during *Pba*-caused infection (Figure 2B,C).

In the necrotic zone, the first sub-fraction of the buffer-extractable fraction included mainly fragments of RG-I (the ratio Rha/GalA ≈ 1), while the second sub-fraction predominantly contained fragments of PGA (the ratio Rha/GalA ≈ 0.25) (Figure 2B). The Gal side chains of RG-I were likely to be shortened in the course of infection since the Gal/Rha ratio in the first sub-fractions were 3.2, 1.8, and 0.7 for the control plants, asymptomatic zone, and necrotic zone, respectively. Thus, in the necrotic zone, relatively high molecular weight (50–400 kDa) RG-I fragments with relatively low Gal substitution were accumulated in the buffer-extractable fraction, indicating that these fragments did not have tight bonds with PCW. PGA fragments accumulated in the buffer-extractable fraction had a relatively low molecular weight (< 50 kDa) (Figure 2A,C). This indicates that in the course of infection, the RG-I is split into larger fragments than PGA.

#### 2.1.2. Polysaccharides of the Ammonium Oxalate Extractable Fraction

**Figure 3:**
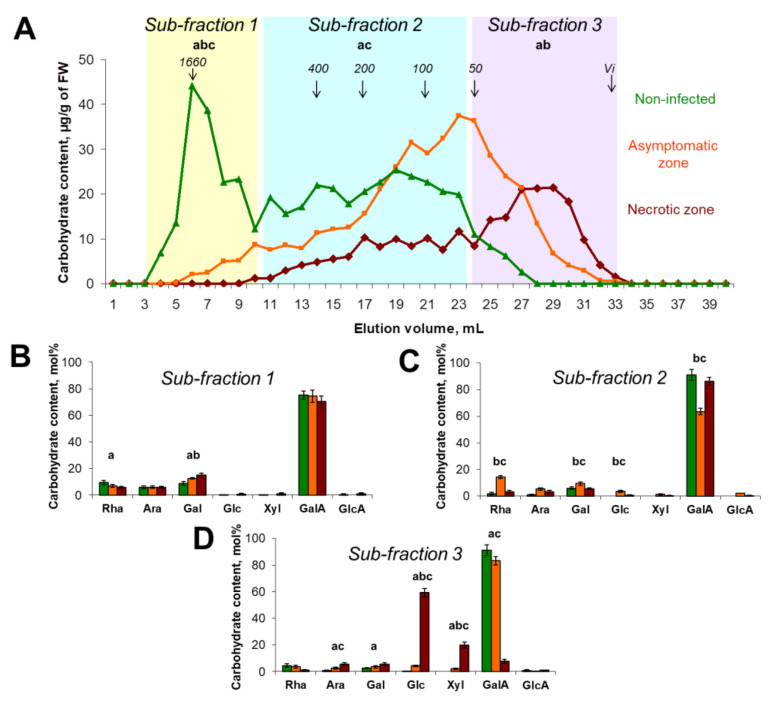
The molecular weight distribution and monosaccharide composition of polysaccharides extracted with 0.5% ammonium oxalate from the plant cell walls isolated from stems of control non-infected (green) or *Pectobacterium atrosepticum*-infected (orange and brown) potato plants: asymptomatic zone (orange) and necrotic zone (brown). (**A**) Elution profiles were obtained using size-exclusion chromatography on a Sepharose CL-4B column; pullulans were used as molecular weight markers; Vi—inclusion volume; values are the means (per g of fresh weight, FW) of three biological replicates. Monosaccharide composition was analyzed in different sub-fractions, i.e., (**B**) sub-fraction 1, (**C**) sub-fraction 2, and (**D**) sub-fraction 3, which are marked on elution profiles following hydrolyzation with trifluoride acetic acid; values are the means ±SD (mol%) of three biological replicates. Small letters a, b, and c point to the significant difference in the carbohydrate content within a particular sub-fraction (panel A) or in mol% content of a particular monosaccharide (panels B–D) (Mann–Whitney two-sided test, *p* < 0.05): a—control vs. necrotic zone; b—control vs. asymptomatic zone; c—asymptomatic zone vs. necrotic zone. Monosaccharide names are abbreviated as follows: Rha—rhamnose, Ara—arabinose, Gal—galactose, Glc—glucose, Xyl—xylose, GalA—galacturonic acid, GlcA—glucuronic acid.

The total content of carbohydrates in the ammonium oxalate-extractable fraction was 2-fold lower in the necrotic zone compared to the asymptomatic zone and control plants (Figure 3A). The content of carbohydrates in this fraction in the asymptomatic zone and control plants did not differ significantly. However, the elution profiles of polysaccharides of this fraction differed for the asymptomatic zone and control plants. A bulk of PCW-bound pectic compounds in control plants was represented by high molecular weight (>1500 kDa) polymers (sub-fraction 1): mostly PGA and also some RG-I, which can be covalently attached to each other, forming large molecules with PGA and RG-I-domains [14]. In the asymptomatic zone, a bulk of PCW-bound pectic compounds was less than 400 kDa (sub-fraction 2) (Figure 3A–C). In the necrotic zone, a bulk of oxalate-extractable polysaccharides was less than 50 kDa (sub-fraction 3). Interestingly, poly/oligomers of sub-fraction 3 in the necrotic zone were predominantly represented not by pectic compounds, but by CLGs, since the major monosaccharides in this fraction (80%) were Glc and Xyl (Figure 3D).

Within the sub-fraction 2 (MW 50-400 kDa), the proportion of RG-I/PGA differed for the necrotic zone, asymptomatic zone, and control plants since the Rha/GalA ratio was 0.04, 0.23, and 0.02, respectively (Figure 3C). At the initial stages of colonization (asymptomatic zone), the Rha/GalA ratio was increased 11-fold compared to control, indicating that the RG-I retained more strongly within the PCW than PGA. At advanced stages of colonization (necrotic zone), when the content of PCW-bound pectic compounds was strongly reduced, the Rha/GalA ratio returned to almost the control values, indicating that both PGA and RG-I were mostly not retained in the PCW because of their degradation (Figure 3A,C). This likely means that during the infection, the breakdown of PGA into low molecular weight products outpaces the degradation of RG-I.

At the asymptomatic stage of colonization, the molecular weight (but not the content) of PCW-bound pectic compounds is decreased compared to that in non-infected plants; however, this does not prevent their retention in the PCW. Within the necrotic zone, the content of PCW-bound pectic compounds is dramatically reduced because of their degradation. Some of the pectin degradation products, in spite of their relatively high molecular weight (<50–400 kDa), were not retained by the PCW and passed into the buffer-extractable fraction (Figure 2A,B).

#### 2.1.3. Polysaccharides of KOH-Extractable Fraction

**Figure 4:**
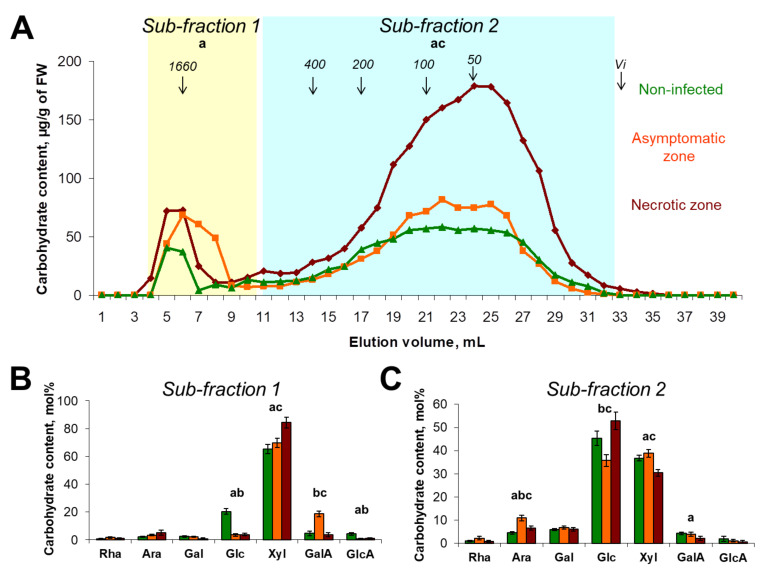
The molecular weight distribution and monosaccharide composition of polysaccharides extracted with 4M potassium hydroxide (KOH) from the plant cell walls isolated from stems of control non-infected (green) or *Pectobacterium atrosepticum*-infected (orange and brown) potato plants: asymptomatic zone (orange) and necrotic zone (brown). (**A**) Elution profiles were obtained using size-exclusion chromatography on a Sepharose CL-4B column; pullulans were used as molecular weight markers; Vi—inclusion volume; values are the means (per g of fresh weight, FW) of three biological replicates. Monosaccharide composition was analyzed in different sub-fractions, i.e., (**B**) sub-fraction 1 and (**C**) sub-fraction 2, which are marked on elution profiles following hydrolyzation with trifluoride acetic acid; values are the means ±SD (mol%) of three biological replicates. Small letters a, b, and c point to the significant difference in the carbohydrate content within a particular sub-fraction (panel A) or in mol% content of a particular monosaccharide (panels B, C) (Mann–Whitney two-sided test, *p* < 0.05): a—control vs. necrotic zone; b—control vs. asymptomatic zone; c—asymptomatic zone vs. necrotic zone. Monosaccharide names are abbreviated as follows: Rha—rhamnose, Ara—arabinose, Gal—galactose, Glc—glucose, Xyl—xylose, GalA—galacturonic acid, GlcA—glucuronic acid.

Two main sub-fractions represented the elution profile of polysaccharides of the KOH-extractable fraction: the minor sub-fraction 1 included high molecular weight polymers of around 1500 kDa, while the major sub-fraction 2 contained the polymers of less than 400 kDa (Figure 4A). The content of polysaccharides in sub-fraction 1 was 2-fold higher in the samples of infected plants (in both necrotic and symptomatic zones) compared to that in control plants. The Xyl/Glc ratio in this sub-fraction was increased during the infection from 3 in the control plants to 20 and 22 in asymptomatic and necrotic zones, respectively, indicating that the proportion of xylan/xyloglucan was increased during the infection within this sub-fraction (Figure 4B). In addition, the elevated content of GalA (19 mol%) was revealed in sub-fraction 1 of the asymptomatic zone compared to that in the necrotic zone and control plants (4 and 5 mol%, respectively). This means that some portion of the high molecular weight pectins is extracted with KOH, but not with ammonium oxalate, and the content of such pectic compounds is increased during the asymptomatic stage of colonization.

The content of carbohydrates in the sub-fraction 2 of the KOH-extractable fraction was around 2.5-fold higher in the necrotic zone compared to that of both the asymptomatic zone and control plants (Figure 4A). The main monosaccharides in this sub-fraction were Glc and Xyl (Figure 4C). The Xyl/Glc ratio differed in the analyzed samples: 0.8, 1.1, and 0.6 in control plants, asymptomatic zone, and necrotic zone, respectively. The increase in the Xyl/Glc ratio in the asymptomatic zone compared to control indicates that during the initial stages of plant colonization by *Pba*, the ratio xylan/xyloglucan was increased within this sub-fraction as well as in the sub-fraction 1. Additionally, in the necrotic zone, the Xyl/Glc ratio was decreased compared to that in the asymptomatic zone and became even lower than that in control plants. Presumably, the amorphous cellulose might contribute to the increase in Glc level in the KOH-extractable fraction of the necrotic zone. However, it should be noted that the increased carbohydrate content in the KOH-extractable fraction of the necrotic zone cannot be attributed to amorphous cellulose only, since the absolute content of Xyl in the sub-fraction 2 was increased 2-fold in the necrotic zone compared to that in control plants. This means that the increase in carbohydrate content in this sub-fraction is at least partially provided by Xyl-containing polysaccharides, namely xylan and/or xyloglucan.

### 2.2. Immunolocalisation of PCW Polysaccharides and Infection-Related Modification of Different Plant Tissues

**Figure 5:**
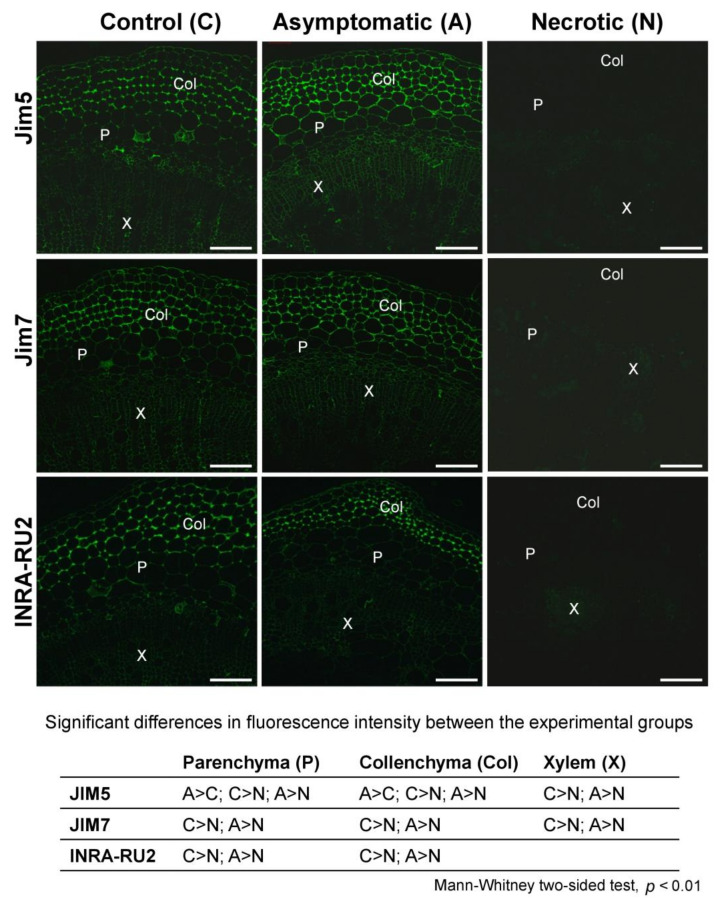
Indirect immunofluorescence detection of polygalacturonan (PGA) and rhamnogalacturonan I (RG-I) epitopes on the stem sections of control non-infected and *Pectobacterium atrosepticum*-infected potato plants; the sections from the infected plants were taken in the asymptomatic and necrotic zones designated in Figure 1. Antibodies Jim5, Jim7, and INRA-RU2 are specific to non-esterified PGA, esterified PGA, and RG-I backbone, respectively. The table located under the photos shows significant differences in immunolabeling intensities for control plants (C), asymptomatic zone (A), and necrotic zone (N) within different tissues: parenchyma (P), collenchyma (Col), and xylem (X). Scale bar: 200 µm. The samples were analyzed in three biological replicates; for each replicate, three photos were analyzed with 10 zones per each photo. Fluorescence intensity values and the details of the statistical analysis are given in the Supplementary Table S1.

The intensity of immunolabeling of epitopes of pectic compounds and cross-linking glycans (CLGs) in non-infected and *Pba*-infected plants (asymptomatic and necrotic zones) was assessed differentially for three tissue types: collenchyma, parenchyma, and xylem. In the necrotic zone, non-esterified PGA (Jim5 antibody), esterified PGA (Jim7 antibody), and RG-I (INRA-RU2 antibody) epitopes were detected at almost background level in all the analyzed tissues in contrast to their intensive labeling in the asymptomatic zone and control plants, indicating that pectic compounds were mostly destroyed at the advanced colonization stage (Figure 5). The de-esterified PGA labeling was more intense in the collenchyma and parenchyma of the asymptomatic zone compared to that in control plants.

**Figure 6:**
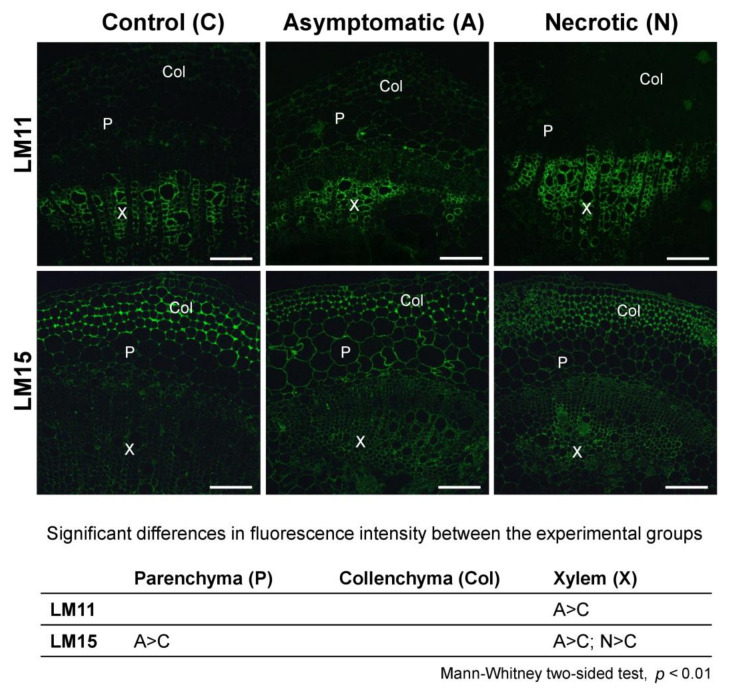
Indirect immunofluorescence detection of xylan and xyloglucan epitopes on the stem sections of control non-infected and *Pectobacterium atrosepticum*-infected potato plants; the sections from the infected plants were taken in the asymptomatic and necrotic zones designated in Figure 1. Antibodies LM11 and LM15 are specific to the backbones of xylan and xyloglucan, respectively. The table located under the photos shows significant differences in immunolabeling intensities for control plants (C), asymptomatic zone (A), and necrotic zone (N) within different tissues: parenchyma (P), collenchyma (Col), and xylem (X). Scale bar: 200 µm. The samples were analyzed in three biological replicates; for each replicate, three photos were analyzed with 10 zones per each photo. Fluorescence intensity values and the details of the statistical analysis are given in the Supplementary Table S1.

The intensity of immunolabeling of xylan epitopes (LM11 antibody) did not differ in all the analyzed variants except that in the xylem, while the labeling in the asymptomatic zone was increased compared to that in control (Figure 6). The labeling of xyloglucan epitopes (LM15 antibody) in the xylem and parenchyma of the asymptomatic zone was increased compared to that in control plants. In the xylem of the necrotic zone, the labeling of xyloglucan was also higher compared to that in control plants.

**Figure 7:**
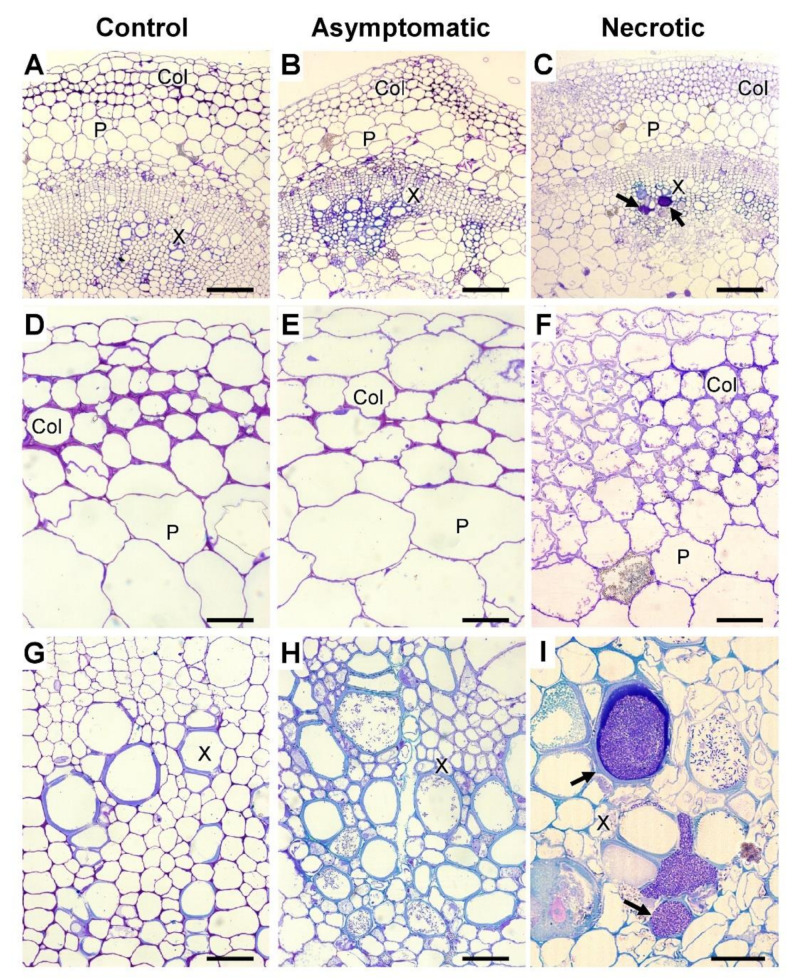
Toluidine blue-stained stem sections of control non-infected (**A**,**D**,**G**) and *Pectobacterium atrosepticum*-infected (**B**,**C**,**E**,**F**,**H**,**I**) potato plants; the sections from the infected plants were taken in the asymptomatic (**B**,**E**,**H**) and necrotic (**C**,**F**,**I**) zones designated in Figure 1. Scale bars: 200 µm for (**A**–**C**), 50 µm for (**D**–**H**), and 25 µm for (**I**). Parenchyma (P), collenchyma (Col), and xylem (X). Arrows in C and I show the bacterial emboli in the primary xylem vessels. (**D**–**F**) are the magnified images of the collenchyma+parenchyma area presented in (**A**–**C**), respectively. (**G**–**I**) are the magnified images of the xylem area presented in (**A**–**C**), respectively. The samples were analyzed in three biological replicates.

To compare the infection-associated destructive modification in different tissues, plant stem sections were analyzed by light microscopy. Although some cell deformation was observed in the parenchyma of *Pba*-infected plants, no dramatic destruction of this tissue was detected in both asymptomatic and necrotic zones (Figure 7). Moreover, the collenchyma underwent severe decay at the advanced stages of colonization (necrotic zone). Within the primary xylem vessels, bacterial emboli were revealed (Figure 7).

### 2.3. Plant and Bacteria PCW-Related Gene Expression

**Figure 8:**
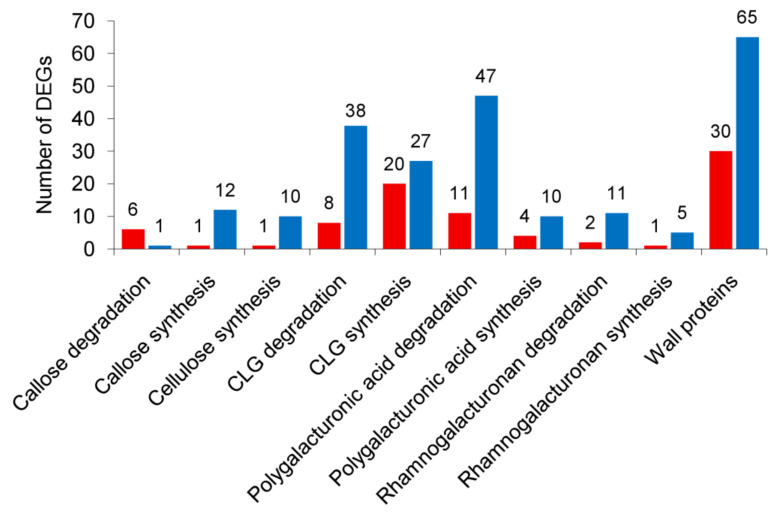
The number of the plant cell wall-related genes differentially expressed in potato plants infected with *Pectobacterium atrosepticum* compared to non-infected plants. Red bars are for the upregulated genes, while blue bars are for the downregulated genes. CLG—cross-linking glycan.

To predict gene products involved in the observed PCW polysaccharide modifications, the expression levels of plant and bacterial PCW-related genes were assessed by RNA-Seq analysis. Among 310 potato PCW-related differentially expressed genes (DEGs), 84 were upregulated and 226 were downregulated during the infection (Figure 8). The details about the expression profile of potato PCW-related genes are given in Supplementary Table S2.

All genes related to cellulose synthesis (encoding cellulose synthase A subunits (CESA) and COBRA-like proteins) were downregulated, except for one upregulated gene encoding COBRA-like protein.

#### 2.3.1. Plant Genes Related to CLG Metabolism

CLG synthesis-related genes were mostly downregulated during the infection; however, some of them, namely those encoding cellulose-synthase-like proteins (CSL), exostosin-like proteins (GT47), and GT61 family glycosyltransferases, as well as TRICHOME BIREFRINGENCE-like (TBL) proteins, were upregulated (Table S2).

**Figure 9:**
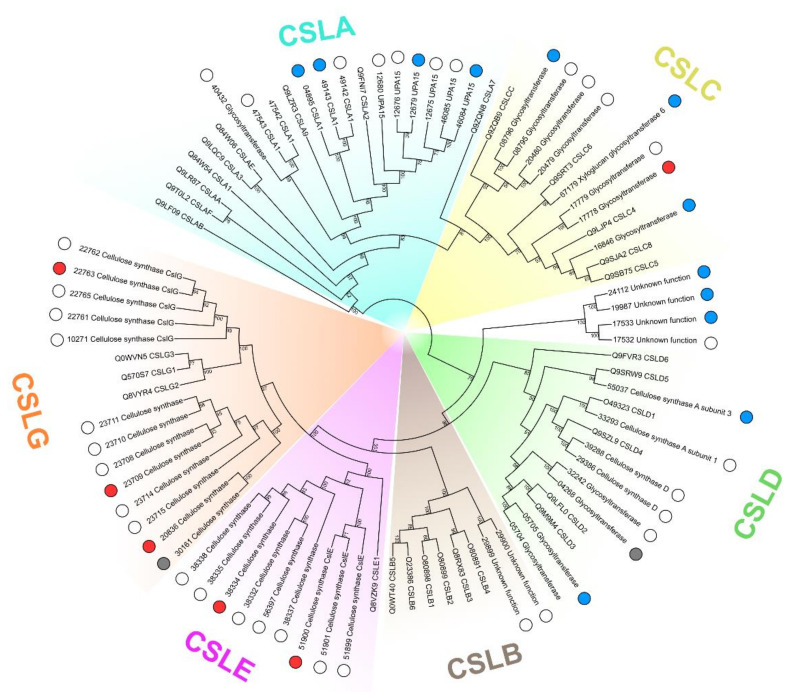
Phylogeny of potato cellulose synthase-like (CSL) proteins (glycosyltransferase 2 family, GT2) and of a reference set of *Arabidopsis thaliana* CSLs. The clades for GT2 functional subgroups are marked in colors. Potato proteins are indicated with circles according to the expression pattern of the corresponding genes in *Pectobacterium atrosepticum*-infected vs. non-infected potato plants: red—upregulated; blue—downregulated; grey—non-DEG; empty—not expressed in our study. The UniProt IDs of *A. thaliana* proteins are given in leaf names. For potato proteins, only parts of PGSC IDs are given; the “PGSC0003DMP4000” symbols, the constant part at the beginning of all presented PGSC protein IDs, were omitted for the sake of brevity.

HMM search and phylogenetic analysis have shown that genes related to the synthesis of glucomannan (CSLA) [15] and xyloglucan (CSLC) [16] backbones were downregulated, except for one CSLC gene that was upregulated (Figure 9). Among the upregulated genes of the CSL subcategory were those encoding CSLG and CSLE, which were predicted to be hemicellulose backbone-forming beta-glycan synthases [17]; however, their exact functions and substrates remain to be determined.

**Figure 10:**
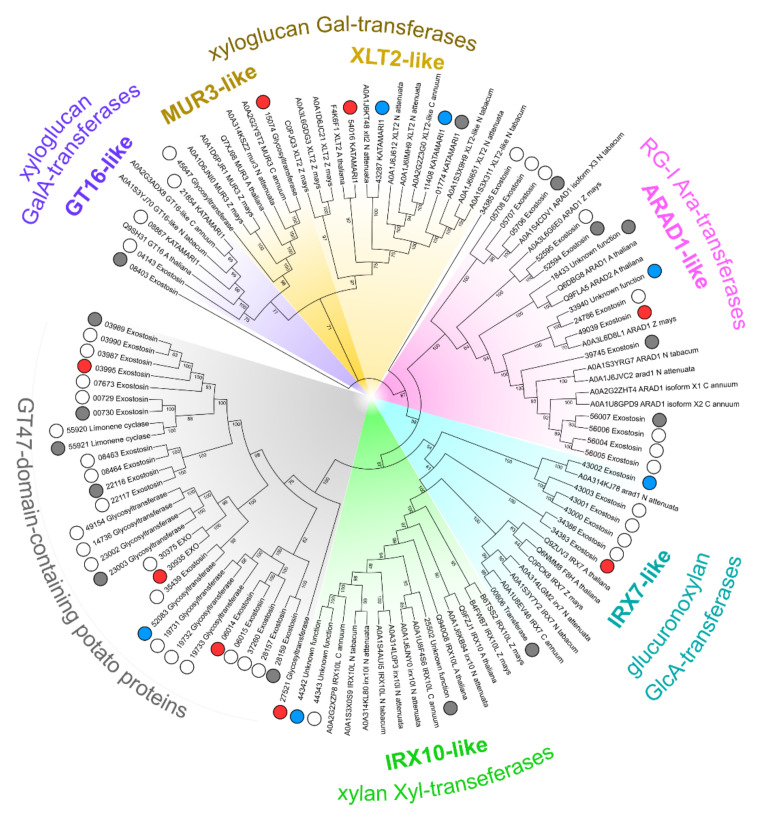
Phylogeny of potato glycosyltransferase 47 family (GT47) proteins and of a reference set of *Arabidopsis thaliana, Capsicum annuum, Nicotiana attenuata, N. tabacum,* and *Zea mays* GT47 proteins. The clades for GT47 functional subgroups are marked in colors. Potato proteins are indicated with circles according to the expression pattern of corresponding genes in *Pectobacterium atrosepticum*-infected vs. non-infected potato plants: red—upregulated; blue—downregulated; grey—non-DEG; empty—not expressed in our study. The UniProt IDs of non-potato species proteins and the corresponding taxa names are given in the leaf names. For potato proteins only parts of PGSC IDs are given; the “PGSC0003DMP4000” symbols, the constant part at the beginning of all presented PGSC protein IDs, were omitted for the sake of brevity.

More than half of DEGs encoding the exostosin family proteins (glycosyltransferase 47, GT47) were upregulated in the infected plants (Table S2). Different evolutionary branches of the enzymes of the GT47 family are involved in different aspects of the synthesis of not only CLGs but also pectin RG-I. Among CLG-synthesizing GT47 enzymes are galacturonosyltransferases (GT16-like) and galactosyltransferases (MUR3, XLT2), yielding side chains of xyloglucan as well as xylosyltransferases (IRX10) and glucuronosyltransferases (IRX7, previously referred to as FRA8) involved in the elongation of the backbone and side chains, respectively, of xylan [18,19,20,21]; the arabinosyltransferases (ARAD1) attach the Ara side chains to the RG-I backbone [22]. Given that the GT47 family enzymes are poorly annotated in potato, to hypothesize on their functions, we reconstructed their phylogeny based on the comparison with well-annotated plant exostosin-like proteins from other species (Figure 10). Two up- and two downregulated DEGs predicted to encode MUR3/XLT2 xyloglucan galactosyltransferases, one up- and one downregulated DEGs for IRX7-like xylan glucuronosyltransferases and one strongly downregulated DEG for IRX10-like xylan xylosyltransferase were revealed in the subcategory of genes for exostosin family proteins. In addition, four upregulated DEGs (including the most strongly upregulated exostosin-encoding DEG PGSC0003DMG400003358) and one downregulated DEG encode the exostosin-like proteins belonging to the potato-specific uncharacterized GT47 proteins, of which the functions could not be inferred from the currently available information (Figure 10).

Along with IRX10 of the GT47 family, the xylan backbone synthesis requires IRX14 and IRX9 proteins belonging to the GT43 family [23]. We performed HMM search of GT43-encoding genes in potato and phylogeny reconstruction of the corresponding enzymes. Among DEGs, two GT47-related genes were revealed (one for IRX14-like protein and one for IRX9-like protein), both of which were downregulated (Figure S1).

To search for the potato genes encoding proteins of the GT61 family involved in the synthesis of xylosyl side chains of xylan [24], HMM/phylogeny analysis was performed. Among six of the revealed GT61-protein-encoding genes, none were downregulated during the infection and three were strongly upregulated (Figure S2). These three upregulated genes (PGSC0003DMG400028330, PGSC0003DMG400023731, and PGSC0003DMG400027288) encode proteins orthologous to *A. thaliana* xylan glycosyltransferase MUCI21 (MUCILAGE-RELATED21) that catalyze the formation of xylosyl side chains on the xylan backbone [24].

A group of 21 DEGs (mostly downregulated) encode TBL (TRICHOME BIREFRINGENCE)-like proteins. Some TBL proteins are O-acetyltransferases specific for xylan (TBL3, TBL22/TBL27/AXY9, TBL29/ESK1, and TBL30–35 [25,26]) and RG-I [27]. By phylogenetic analysis we have shown that three up- and ten downregulated DEGs encode TBLs that are probably active towards xylan and three downregulated—towards RG-I (Figure S3).

Thus, in terms of gene expression, the alterations in CLG synthesis took place during *Pba*-caused infection. Many genes related to the synthesis of xyloglucan and xylan backbones were downregulated, and only one of them (for CSLC) was upregulated. Additionally, genes related to xylosyl side chain formation on the xylan backbone were strongly upregulated, while genes related to xylan acetylation were mostly downregulated. Based on gene expression, we cannot predict whether the repression or induction of xyloglucan side chain synthesis took place during the infection; however, given that both up- and downregulated genes related to the synthesis of xyloglucan side chains were revealed, the de novo synthesized (during the infection) xyloglucan might have structural peculiarities compared to the pre-synthesized one.

As for the CLG degradation-related genes, 46 DEGs encoding endoglucanases, mannosidases, xylosidases, xylanases, and xyloglucan endotransglycosylases/hydrolases (XTH) were revealed. Most of them were downregulated. However, eight genes were strongly upregulated: four for endoglucanases, one for endoxylanase Xyn1, and three for XTH (Table S2).

#### 2.3.2. Plant Genes Related to Pectin Metabolism

The synthesis of PGA seemed to be repressed during the infection, since 10 of 14 DEGs for galacturonosyltransferases were strongly downregulated, while 4 of them were upregulated only slightly (Table S2). The revealed candidate RG-I-encoding gene (cognate to RG-I rhamnosyltransferase gene of *A. thaliana*, [28]) was also downregulated, as well as three genes for the predicted TBL10-like O-acetyltransferases presumably involved in RG-I acetylation (see above).

As for the PGA degradation-related genes, all nine DEGs for PGA-lyases were downregulated. DEGs for polygalacturonases were also mostly downregulated (15 of 18); however, three of them were upregulated more than 10-fold. RG-I-degradation-related DEGs included 10 β-galactosidase-encoding genes, all of which were downregulated, as well as three RG-I lyase-encoding genes, two of which were strongly upregulated up to a level of log_2_FC ≈ 9 (Table S2).

#### 2.3.3. Plant Genes Encoding Other Cell Wall Proteins

A total of 18, 20, 25, and 12 DEGs for expansin-like, extensin-like, arabinogalactan-like, and chitinase-like proteins, respectively, were revealed (Table S2). Six up- and 12 downregulated genes for expansin-like proteins were found. Moreover, all of the revealed DEGs for expansin-like B (EXLB) proteins were upregulated, while DEGs for expansin A subfamily (EXPA) proteins were mostly (except for two genes) downregulated. DEGs encoding extensin-like proteins were equally (10:10) distributed among up- and downregulated ones. Almost all DEGs encoding arabinogalactan proteins (22 of 25) were downregulated, while chitinase-like protein-encoding DEGs were mostly (7 of 12) upregulated.

#### 2.3.4. *Pba* Genes Encoding Plant Cell Wall Degrading Enzymes (PCWDEs)

Among 16 *Pba* genes encoding the enzymes that cleave glycosidic bonds in PGA, 9 were upregulated and 2 were downregulated (Table S3). Genes for polysaccharide esterases were either non-DEGs or downregulated. Six of eight genes related to RG-I degradation were upregulated; three of them encode the enzymes that cleave RG-I backbone (RG-I lyase/hydrolase), while the others three encode the Gal side chains (β-galactosidase and endo-β-1,4-galactanase). In addition, we revealed three genes encoding the enzymes/proteins for degradation of non-pectic polymers or bonds between polymers: expansin-like protein [29], incorrectly annotated as cellulase ECA2220, endoglucanase, and putative xylosidase/arabinosidase. For details, see Table S3.

## 3. Discussion

### 3.1. The Modification of Pectic Compounds in Potato Plants during Pba-Caused Infection

PCW-bound pectic compounds were almost completely degraded in potato plants in the course of *Pba*-caused infection (Figure 2, Figure 3). This was not a surprise, since the representatives of Soft Rot *Pectobacteriaceae* (SRP), including *Pba*, are well-known to produce a large set of enzymes that cleave glycosidic bonds in pectins [30,31,32]. We have shown that the initiation of pectin modification starts in the asymptomatic zone, where huge pectic molecules of >1500 kDa present in control plants are disintegrated to fragments of <400 kDa that are still retained in the PCW (Figure 2). Interestingly, although we did not reveal an increase (as well as the decrease) in the total PCW-bound pectin content in the asymptomatic zone compared to control plants and pectin-biosynthetic genes were not upregulated during the infection, the intensity of immunolabeling of the de-esterified PGA was higher in the asymptomatic zone compared to control plants (Figure 5). Presumably, this can be explained by the increased availability of epitopes for antibodies in the fragmented pectic molecules (which content is higher in the asymptomatic zone) compared to the “intact” huge pectin molecules (present only in control plants).

The modification of pectic compounds during the infection was likely to be implemented by gene products of both the host plant and the pathogen. Many *Pba* genes encoding the enzymes for degradation of PGA and RG-I were upregulated in infected plants compared to the in vitro culture (Table S3). Pectin degradation-related genes of potato plants were regulated differentially during the infection: PGA-lyases were all downregulated, while among the genes encoding polygalacturonases and RG-I-lyases, both up- and downregulated ones were revealed (Table S2). Interestingly, a similar expression pattern of pectin degradation-related genes was observed in *Pba*-infected tobacco plants [13]. One of the most infection-upregulated tobacco genes, LOC107816998, encoding the RG-I-lyase had the similar gene in potato (PGSC0003DMG400014600) which was also among the most upregulated ones in the course of *Pba*-caused infection (Table S4). However, although in both plant species the infection-upregulated polygalacturonase genes were revealed, a set of these genes differed in potato and tobacco plants.

In fact, we mentioned that the similar genes of potato (this study) and tobacco [13] had mostly similar expression patterns during *Pba*-caused infection compared to non-infected plants (Table S4). This indicates that the transcriptional responses of experimental and natural hosts to *Pba*-caused infection are similar in general (with some unique features) at least for PCW-related genes. Full information on the comparison of similar PCW-related gene expression is given in the supplementary Table S4 (note that in this table, a particular gene can be mentioned several times, since one potato gene may correspond to several tobacco genes and vice versa). *Pba* genes encoding PCWDEs also had mostly similar expression patterns irrespective of whether the experimental or natural host was colonized by the bacteria (Table S3).

The immunolocalization of epitopes of pectic compounds has shown that their content within the necrotic zone was reduced (compared to control) similarly in collenchyma and parenchyma (Figure 5). However, we presume that either the degradation of pectins was more pronounced in the collenchyma than in parenchyma or a similar level of pectin degradation differentially influenced the integrity of collenchyma and parenchyma. This suggestion was made since the collenchyma underwent much more pronounced destruction during the infection than parenchyma (Figure 7). In tobacco plants, whose stems lack the outer collenchyma layer, *Pba*-caused disease is associated with a dramatic disintegration of parenchyma [12,33]. In potato, the core parenchyma, although it underwent some deformation, looked relatively “intact” compared to collenchyma. Thus, our study shows that in the potato plant stems, the collenchyma is the most “vulnerable” tissue during *Pba*-caused infection.

Although the degradation of pectic compounds proceeded intensively during the infection, some products of RG-I degradation had a relatively high molecular weight (50–400 kDa) and were not retained in the PCW eluting in the buffer extractable fraction (Figure 2). These RG-I fragments had low Gal substitution, indicating that the Gal side chains were cleaved from the RG-I backbone in the course of infection. Presumably, it was *Pba* enzymes that cleaved Gal side chains of RG-I since three *Pba* β-galactosidase/endo-β-1,4-galactanase genes were upregulated in planta (Table S3) and none of plant β-galactosidase genes were upregulated during the infection, while most of them were downregulated (Table S2). Such kinds of low-Gal-substituted, 50–400 kDa RG-I fragments were previously shown to prime the assemblage of bacterial emboli, forming an initial extracellular matrix that was further substituted by *Pba* exopolysaccharides [10,34]. It should be noted that the bacterial polysaccharides were unlikely to contribute to the obtained data: first, we have previously shown that the portion of bacterial polysaccharides is negligible in the whole polysaccharide pool of the infected plant [10], and second, exopolysaccharides of *Pba* have the specific content and proportion of monosaccharides [34], and thus, their contribution to the whole carbohydrate pool would be evident if this was the case.

Given that in *Pba*-infected potato plants the “primers” (PCW-non bound RG-I fragments of 50–400 kDa with low Gal substitution) for the formation of bacterial emboli were accumulated, we tested whether these structures were formed not only in the experimental host but in the natural host also. We have found that indeed bacterial emboli were formed in potato plants in the primary xylem vessels (Figure 7). Although we cannot assess the number of bacterial emboli within a plant, it was evident that the frequency of occurrence of bacterial emboli in potato plants was much lower than in tobacco plants. It is possible that the intensive formation of bacterial emboli in the natural host is timed to the particular host plant developmental stage or requires additional external or internal factors. Moreover, the increased formation of bacterial emboli in tobacco could be promoted by growing the plants under sterile in vitro conditions [10,12,33], while the analyzed potato plants were grown under non-sterile conditions in the soil (this study). The possibility that the bacterial emboli are indeed formed less intensively in the natural host than in the experimental one also cannot be excluded. Nevertheless, the fundamental possibility of the formation of *Pba* bacterial emboli in the natural host potato was demonstrated in our study.

### 3.2. The Modification of CLGs in Potato Plants during Pba-Caused Infection

Our study shows for the first time that the infections caused by the representative of the SRP touch not only pectic compounds but also CLGs. The content of carbohydrates in the KOH-extractable PCW fraction was increased at the advanced stages of plant colonization (necrotic zone) primarily due to Glc- and Xyl-containing polysaccharides (Figure 4). Higher Glc level, in this case, may be associated (at least partially) with an increase in the proportion of amorphous cellulose during the infection, a portion of which could be extracted by KOH. However, given that an increase in Xyl level was also observed in the KOH-extractable fraction, we have presumed that the synthesis of either xylan or xyloglucan or both was enhanced during the infection. The increase in xylan/xyloglucan immunolabeling was previously observed in potato plants during the infection caused by the Potato virus Y (PVY); the resistance to PVY associated with the hypersensitive response and PCW reinforcement was not coupled with the increased xylan/xyloglucan immunolabeling [35]. Therefore, the increase of the CLG level can be presumed to be an attribute of infection-associated PCW loosening.

Among genes related to the syntheses of xylan and xyloglucan backbones, only one was upregulated during *Pba*-caused infection (Table S2, Figure 9, Figure S1). In turn, genes encoding the enzymes that form xylosyl side chains on the xylan backbone were strongly upregulated (Table S2, Figure S2). These data were in accordance with the increased level of Xyl in the KOH-extractable fraction of the necrotic zone. This also explains why we did not reveal the enhanced immunolabeling of xylan in the necrotic zone (Figure 6): the LM11 antibody is specific to non-substituted xylan backbone (or Ara/GlcA-substituted) but not to Xyl-substituted [36]. In addition, genes related to xylan acetylation (TBL family proteins) were mostly downregulated (Table S2, Figure S3).

Thus, it can be presumed that in the *Pba*-infected potato plants, the “modified” xylan, with lower acetylation and a higher level of xylosyl side chains compared to that in control plants, was synthesized. Such structural features are known to affect xylan properties. Compared to acetylated xylans, deacetylated xylans form more loose and highly hydrated layers on cellulose microfibrils, resulting in the reduction of PCW rigidity. Acetyl groups are considered to impede the enzymatic digestion of xylan and the overexpression of xylan-specific acetylesterases was shown to increase pathogen resistance [37,38,39,40]. In turn, the increased branching of xylan with Xyl residues was proposed to be necessary for their interactions with pectic compounds [24]. Thus, it is reasonable to presume that the alterations in structural characteristics of xylan molecules may influence plant-*Pba* interactions and the synthesis of the “modified” xylan molecules in the course of the infection is not a random event.

As for the synthesis of side chains of xyloglucan, among the corresponding genes, both up- and downregulated ones were revealed. Moreover, genes for the enzymes that attach xylosyl side chains to the xyloglucan backbone were either non-DEGs or downregulated. The immunolabeling of xyloglucan epitopes was increased in the infected plants (in both necrotic and asymptomatic zones) compared to non-infected plants, indicating that either the increase in the xyloglucan level or the modification of its structure (or of the structure of other polymers) occurred during the infection, making it more available for binding the antibodies. However, based on the obtained data, we cannot propose what kinds of modifications (if any) of xyloglucan side chains took place during the infection.

The most known modification of xyloglucan that significantly influences the properties of the PCW is its transglycosylation by XTHs. Such modification, along with the action of expansins, provides PCW loosening and stretching, e.g., during cell extension growth and fruit ripening [41,42]. In our previous study on tobacco plants, based on RNA-Seq data, we have proposed that the observed upregulation of XTH- and expansin-encoding genes during *Pba*-caused infection might promote the release of RG-I fragments from the PCW and facilitate PCW degradation by increasing the availability of polymers to the corresponding enzymes [13]. In *Pba*-infected potato plants, the infection-upregulated XTH- and expansin-encoding genes were also revealed (this study). A set of the upregulated XTH-encoding genes was mostly different in tobacco and potato, while the expression profiles of various expansin-encoding genes were similar in two plant species after *Pba* infection: genes for expansin-like B proteins were upregulated, while most of the expasin A-encoding ones were downregulated (Table S4). Therefore, the revealed upregulation of XTH- and expansin-encoding genes in *Pba*-infected potato plants strengthens our hypothesis on the role of these enzymes/proteins in plant susceptibility to this pathogen; additionally, it indicates that the modification of CLGs (and their interaction with cellulose) is associated with *Pba*-caused disease.

## 4. Materials and Methods

### 4.1. Growth Conditions for Plants and Bacteria, Plant Inoculation, and Sample Collection

Sterile virus-free potato plants (*Solanum tuberosum* cv. Kondor) were axenically grown on Murashige and Skoog medium in tubes in a growth chamber with a 16-h light/8-h dark cycle photoperiod. Apical stem parts (~2 cm) were cut and rooted on sterile vermiculite (Peter Peat, Russia) for 14 days and then planted into the soil (Technoexport, Russia) in 300 mL pots. Four weeks after planting into the soil, plants were infected (or not infected) by *Pectobacterium atrosepticum* SCRI1043 (*Pba*) and transferred to glass chambers 40 cm × 60 cm × 60 cm to provide high humidity.

*Pba* was cultured in Luria-Bertani (LB) broth [43] on a rotary shaker (180 rpm) at 28 °C until the early stationary phase (~2 × 10^9^ colony-forming units (CFU/mL)). For plant inoculation, bacterial cells were washed twice with and suspended in sterile 10 mM MgSO_4_ up to a density of ~2 × 10^7^ CFU/mL. Ten µl of the obtained bacterial suspension or sterile 10 mM MgSO_4_ was injected into the middle parts of the plant stems using a syringe. Three days after plant inoculation, the samples were collected for the analyses. For the infected plants, two zones were collected and analyzed separately: (1) the necrotic zone located around the inoculation point and (2) the asymptomatic zone located 1 cm below the necrotic zone (Figure 1). For non-infected plants, the stem area located 1 cm below the zone of MgSO_4_ infiltration was collected. For RNA-Seq analysis, only asymptomatic zones of the infected plants and stem sections of control plants were analyzed.

### 4.2. RNA Extraction and cDNA Library Preparation and Sequencing

Plant material was ground in liquid nitrogen and early stationary phase *Pba* cells were harvested (8000 g, 5 min, room temperature). The obtained powder or *Pba* cell pellets were resuspended in 1 mL of ExtractRNA Reagent (Evrogen, Moscow, Russia) and the subsequent procedures were performed according to the manufacturer’s instructions. Residual DNA was eliminated using a DNA-free kit (Life Technologies, Carlsbad, California, USA). RNA quantity and quality were analyzed using a Qubit fluorimeter (Life Technologies, Carlsbad, California, USA) and Qsep100 DNA Analyzer (Bioptic, New Taipei City, Taiwan), respectively.

For mRNA enrichment, total RNA (3 μg) was processed using Ribo-Zero rRNA Removal Kit (Plant) (Illumina, San Diego, California, USA) and then Ribo-Zero rRNA Removal Kit (Gram-Negative Bacteria) (Illumina, San Diego, California, USA). cDNA libraries were prepared using a NEBNext Ultra II Directional RNA Library Prep Kit (New England Biolabs, Ipswich, Massachusetts, USA). Qualitative and quantitative evaluation of the RNA and cDNA libraries was performed with a Qubit fluorometer (Invitrogen, USA) and 2100 BioAnalyzer (Agilent Technologies, Santa Clara, California, USA). The validation of the cDNA libraries before sequencing was performed using a CFX96 Touch Real-Time PCR Detection System (Bio-Rad, Hercules, California, USA). Libraries were sequenced in three biological replicates. Sequencing was conducted on Illumina HiSeq 2500 (Illumina, San Diego, California, USA) at the Joint KFU–Riken Laboratory, Kazan Federal University (Kazan, Russia).

### 4.3. RNA-Seq Data Processing and Analysis of Differential Expression

Raw reads generated in this study are available at the NCBI BioProject, accession number PRJNA741058. The quality of the reads was assessed using FastQC (http://www.bioinformatics.babraham.ac.uk/projects/fastqc/, accessed on 31 May 2021). Reads with a q-score < 30 and rRNA-corresponding reads were filtered out using Trimmomatic and SortMeRNA, respectively [44,45]. Pseudo-alignment and quantification of filtered reads were carried out using kallisto [46] with default parameters and reference sequences of *S. tuberosum* and *Pba*. To create the optimal reference for read pseudo-alignment, the EvidentialGene package (https://sourceforge.net/projects/evidentialgene/, accessed on 31 May 2021) was used with default parameters to reduce the redundancy of potato protein-coding sequences obtained from *S. tuberosum* DM Genome Annotation v3.4 [47]. Coding sequences of the *Pba* genome, available at Ensembl genomes (ftp://ftp.ensemblgenomes.org/pub/bacteria/release-35/fasta/bacteria_68_collection/pectobacterium_atrosepticum/cds/Pectobacterium_atrosepticum.ASM69646v1.cds.all.fa.gz, accessed on 31 May 2021), were used as reference. The edgeR package [48] was used to reveal differentially expressed genes (DEGs). Genes that had TMM-normalized read counts per million (CPM) values ≥ 1 in all replicates within at least one of the experimental conditions were considered to be expressed in our study. Genes with |log_2_FC| < 1 and FDR < 0.05 were considered to be DEGs. The functional annotations were assigned to the *Pba* and potato DEGs, as described earlier [13,33].

### 4.4. Phylogenetic Analysis

The phylogenetic analysis of the selected potato protein families—glycosyltransferases (GT) 2, 43, 47, and 61 and Trichome birefringence-like (TBL) proteins—was carried out to split them into functional groups according to the orthology with the corresponding reference proteins of other plant species. All potato proteins of the listed GT families were extracted from the PGSC v3.4 reference proteome using HMMER v.3.3.2 (http://hmmer.org/, accessed on 31 May 2021) and R software. The following Pfam GT domains were used for HMM search: GT2—PF00535, PF10111, PF13632, PF13704, PF13712, and PF03552; GT34—PF05637; GT47—PF03016; and GT61—PF04577. TBL proteins were found in the potato proteome using BLAST against *Nicotiana tabacum* and *Arabidopsis thaliana* TBL proteins. Only TBL encoded by potato DEGs were used for phylogenetic analysis. The non-potato reference sequences for every listed family were obtained from the UniProt database (the corresponding IDs and species names are given on the cladograms) and added to each set of potato proteins.

Multiple sequence alignment was carried out for each obtained set of proteins using web-based MUSCLE by EMBL-EBI [49] with default settings. These alignments were used to construct dendrograms by means of IQ-TREE v.2.0.3 software with 10,000 replicates bootstrap support and automated selection of the best-fit model of sequence evolution for each alignment [50]. The dendrograms were visualized with iTOL v.6 (https://itol.embl.de/, accessed on 31 May 2021) and edited in Inkscape (https://inkscape.org, accessed on 31 May 2021). Cellulose synthase A (CESA) orthologs were removed from the GT2 dendrograms, so only Cellulose synthase-like (CSL) proteins remained on the tree.

### 4.5. Plant Cell Wall (PCW) Isolation and Fractionation

Plant tissues were ground in liquid nitrogen in mortars. Five volumes of 50 mM Na/K-phosphate buffer (pH 7.0) were added to the obtained powders and the grinding continued until the full thawing of the samples. The obtained homogenates were centrifuged for 10 min at 10,000× *g* and 4 °C. Then, the supernatants containing buffer-extractable fractions were collected, incubated at 100 °C for 10 min and centrifuged for 10 min at 10,000× *g* and 4 °C. The buffer-extractable polysaccharides were precipitated overnight by four volumes of 96% ethanol. Then, the precipitates were harvested, washed three times with 80% ethanol, and diluted in deionized water.

The pellets remained after the separation of buffer-extractable fraction were suspended in 80% ethanol, incubated at 100 °C for 10 min, and harvested (10 min at 10,000× *g* and 4 °C). The pellets were washed with 80% ethanol, 50 mM Na/K-phosphate buffer, and 100% acetone three times each. For starch depletion, samples were treated twice with glucoamylase (1 mg/g of fresh plant material) (Sigma, Saint Louis, MO, USA) in phosphate buffer supplemented with 0.02% NaN_3_ for 18–20 h each at 37 °C. The absence of starch in the samples was proved by KI-I_2_ staining. After starch depletion, samples were washed with deionized water and acetone three times each and dried.

For the extraction of PCW-bound pectic compounds, the obtained PCW pellets were suspended in 0.5% ammonium oxalate (pH 5.0) and incubated at 100 °C for an hour. After the centrifugation (10 min at 10,000× *g* and 4 °C), the supernatants were collected and ammonium oxalate was again added to the pellets followed by incubation at 100 °C for an hour. The supernatants obtained after both ammonium oxalate extractions were combined, desalted by passage through a Sephadex G-10 column (19 × 400 mm, Pharmacia, Stockholm, Sweden), dried, and diluted in deionized water.

For the extraction of CLGs, the water-washed PCW pellets remained after ammonium oxalate extraction were suspended in 4 M KOH with 3% H_3_BO_4_ and incubated 6 h at 25 °C. The supernatants were collected and KOH with 3% H_3_BO_4_ was, again, added to the pellets followed by incubation at 25 °C for 6h. The supernatants obtained after both KOH extractions were combined, neutralized by adding acetic acid to pH 7.0, desalted by passage through a Sephadex G-25 column (19 × 400 mm, Sigma, Saint Louis, MO, USA), dried, and diluted in deionized water.

### 4.6. Molecular Weight Distribution Analysis

Size-exclusion chromatography of polysaccharides was carried out on a Sepharose CL-4B column (1.2 × 40 cm, Pharmacia, Stockholm, Sweden) using 0.01 M of pyridine/acetic acid solution, pH 4.5, a flow rate of 0.25 mL/min, and a fraction volume of 1.0 mL. Pullulan samples of 1660, 380, 186, 100, and 48 kDa (Showa Denko, Tokyo, Japan) with a low index of polydispersity (1.09–1.19) were used as standards for column calibration. The carbohydrate content in each fraction was measured by phenol-sulfuric acid assay [51].

### 4.7. Monosaccharide Analysis

Polysaccharides of sub-fractions obtained after size-exclusion chromatography were hydrolyzed with 2 M trifluoride acetic acid (TFA) (Sigma, Saint Louis, MO, USA) at 120 °C for 1 h and dried in a stream of air at 60 °C. Monosaccharide analysis was carried out by high performance anion-exchange chromatography (HPAEC) on CarboPac PA-1 column (4 × 250 mm, Dionex, Sunnyvale, CA, USA), using pulse-amperometric detection (PAD, Dionex, Sunnyvale, CA, USA). Triple waveform B for the analysis of carbohydrates by ion chromatography using a gold working electrode was applied. Eluents: A—0.015 M NaOH; B—1 M NaOAc in 0.1 M NaOH. The column was equilibrated with eluent A—100%. The sample was eluted with the following linear gradients: 0–20 min A–100%; 20–21 min A—90%, B—10%; 21–31 min A—70%, B—30%; flow rate 1 mL/min at 30 °C. Monosaccharide standards (Merck, Darmstadt, Germany) were treated with 2 M TFA at 120 °C, 1 h before they were used for calibration. Data were analyzed using PeakNet software.

The analysis of plant cell wall polysaccharides using size-exclusion chromatography and determination of monosaccharide content were performed in three biological replicates.

### 4.8. Microscopy and Immunocytochemistry

Stem sections designated in Figure 1 of control or *Pba*-infected potato plants were fixed in a mixture of 2% paraformaldehyde and 0.5% glutaraldehyde prepared in 0.1 M phosphate buffer (pH 7.2) for 4 h at 4 °C, then dehydrated in a graded aqueous ethanol series, progressively infiltrated with LR White resin (Medium Grade Acrylic Resin; Ted Pella, Redding, California, USA), and after that, embedded in 100% resin in Beem capsules. The resin was polymerized at 60 °C for 24 h. Semi-thin sections (1 μm thick) were prepared using a glass knife on a LKB 8800 ultramicrotome (LKB Instruments, Mount Waverley, Australia). The sections were stained with 0.5% (w/v) toluidine blue in water for 5 min and examined using a laser confocal microscope (LSM 510 Meta; Carl Zeiss, Oberkochen, Germany) under transmitted light. The images were acquired with an AxioCam HRs camera (Carl Zeiss, Oberkochen, Germany).

The immunolabeling was carried out using antibodies INRA-RU2 (RG-I backbone [52], JIM5 and JIM7 (non-esterified and esterified PGA, respectively [53]), LM11 (xylan backbone [36]), and LM15 (xyloglucan backbone, [54]). The sections collected on silane-coated microscope slides were (1) pre-incubated in Na-phosphate buffered saline (PBS), pH 7.4, containing 3% (w/v) bovine serum albumin (BSA) for 1 h to block non-specific labeling; (2) incubated with the primary monoclonal antibodies diluted in 0.1 M PBS with 0.06% (w/v) BSA for 1 h; and (3) incubated with secondary antibody linked to fluorescein isothiocyanate (FITC, Sigma-Aldrich, USA) diluted 1:100 in 0.1 M PBS with 0.06% (w/v) BSA for 1 h. JIM5 and JIM7 were diluted 1:10; LM11, LM15, and INRA-RU2 were diluted 1:5. Goat anti-mouse secondary antibodies were used against INRA-RU2 antibodies, while goat anti-rat was used against all other used primary antibodies. Primary antibodies were omitted in control experiments. The sections were examined using a laser confocal fluorescence microscope (LSM 510 Meta; Carl Zeiss, Oberkochen, Germany) with blue excitation from an HBO mercury vapor lamp (Carl Zeiss, Oberkochen, Germany) using a 450–490 nm bandpass excitation filter, a 510 nm color splitter, and a 515–565 nm barrier filter. Samples were photographed with an AxioCam HRs camera (Carl Zeiss, Oberkochen, Germany). The exposure time and camera settings were maintained constant for each antibody.

The fluorescence intensity was measured on ×10 magnification images using ImageJ2 Fiji software1. After background removal by subtracting background function, the mean signal intensity was determined in the square of fixed size placed on the particular tissue. The samples were analyzed in three biological replicates; for each replicate, three photos were analyzed, with 10 zones per each photo.

## 5. Conclusions

Our study shows the specific modification of RG-I, yielding high molecular weight, low-Gal-substituted, PCW-non-bound fragments (the “primers” for the formation of bacterial emboli), which is implemented in a similar way in *Pba*-infected potato and tobacco plants. We demonstrated that the bacterial emboli are formed by *Pba* not only in the experimental host (tobacco) but also in the natural host (potato). Collenchyma was shown to be the most “vulnerable” to *Pba* tissue among the potato stem tissues. The infection caused by the representative of the Soft Rot *Pectobacteriaceae* was shown to affect not only pectic compounds but also CLGs; the content of CLGs was increased in *Pba*-infected plants compared to non-infected ones. Based on gene expression and monosaccharide composition analyses, the ramified xylan with xylosyl side chains was proposed to be accumulated in *Pba*-infected plants. To the best of our knowledge, this is the first study that considers the changes in the entire set of matrix PCW polysaccharides in the course of a plant infectious disease.

## Data Availability

RNA-Seq reads are available at PRJNA741058.

## Supplementary Materials

The following are available online at https://www.mdpi.com/article/10.3390/plants10071407/s1: Supplementary Figures (a PDF file containing Figures S1–S3 with captions and legends); Supplementary Tables: Table S1: The fluorescence values and the details on statistical analysis of immunolabeling intensities of PCW matrix polysaccharides in control and *P. atrosepticum*-infected potato plants (an Excel workbook), Table S2: The list of categorized PCW-related DEGs of *S. tuberosum* (an Excel workbook), Table S3: The list of categorized PCWDE-encoding DEGs of *P. atrosepticum* (an Excel workbook), Table S4: The comparison of the expression of the similar PCW-related genes regulated in *S. tuberosum* and *N. tabacum* during *P. atrosepticum*-caused typical infection (an Excel workbook).
