# Supplementary material for: The Modification of Plant Cell Wall Polysaccharides in Potato Plants during Pectobacterium atrosepticum-Caused Infection
Source: Plants (Basel). 2021 Jul 9;10(7):1407. doi: 10.3390/plants10071407 (PMC8309280; doi:10.3390/plants10071407)
Supplement: Supplementary file 1 [file plants-10-01407-s001.zip › Supplementary Figures S1-S3.pdf]

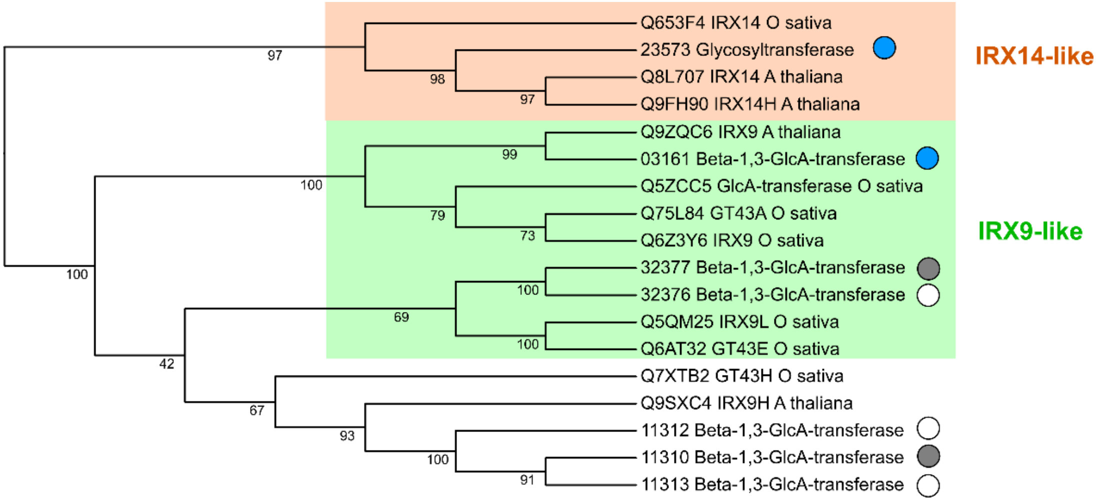

Figure S1. Phylogeny of potato glycosyltransferase 43 family (GT43) proteins and of a reference set of *Arabidopsis thaliana* and *Oryza sativa* GT43 proteins. The clades for GT43 functional subgroups are marked in colors. Potato proteins are indicated with circles according to the expression pattern of corresponding genes in *Pectobacterium atrosepticum*-infected vs. non-infected potato plants: red—upregulated; blue—downregulated; grey—non-DEG; empty—not expressed in our study. The UniProt IDs of non-potato species proteins and the corresponding taxa names are given in the leaf names. For potato proteins only parts of PGSC IDs are given; the "PGSC0003DMP4000" symbols, the constant part in the beginning of all presented PGSC protein IDs, were omitted for the sake of brevity.

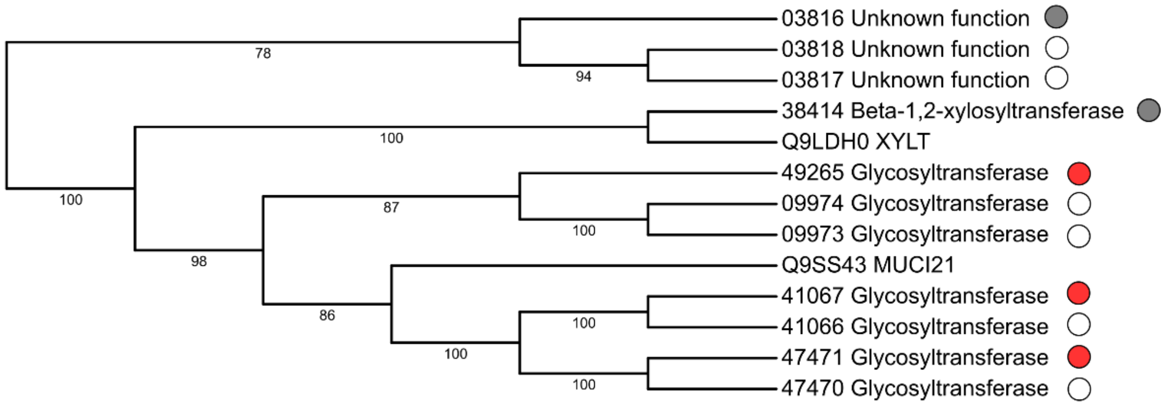

Figure S2. Phylogeny of potato glycosyltransferase 61 family (GT61) proteins and of a reference set of *Arabidopsis thaliana* GT61 proteins. Potato proteins are indicated with circles according to the expression pattern of corresponding genes in *Pectobacterium atrosepticum*-infected vs. non-infected potato plants: red—upregulated; blue—downregulated; grey—non-DEG; empty—not expressed in our study. The UniProt IDs of *A. thaliana* proteins are given in the leaf names. For potato proteins only parts of PGSC IDs are given; the "PGSC0003DMP4000" symbols, the constant part in the beginning of all presented PGSC protein IDs, were omitted for the sake of brevity.

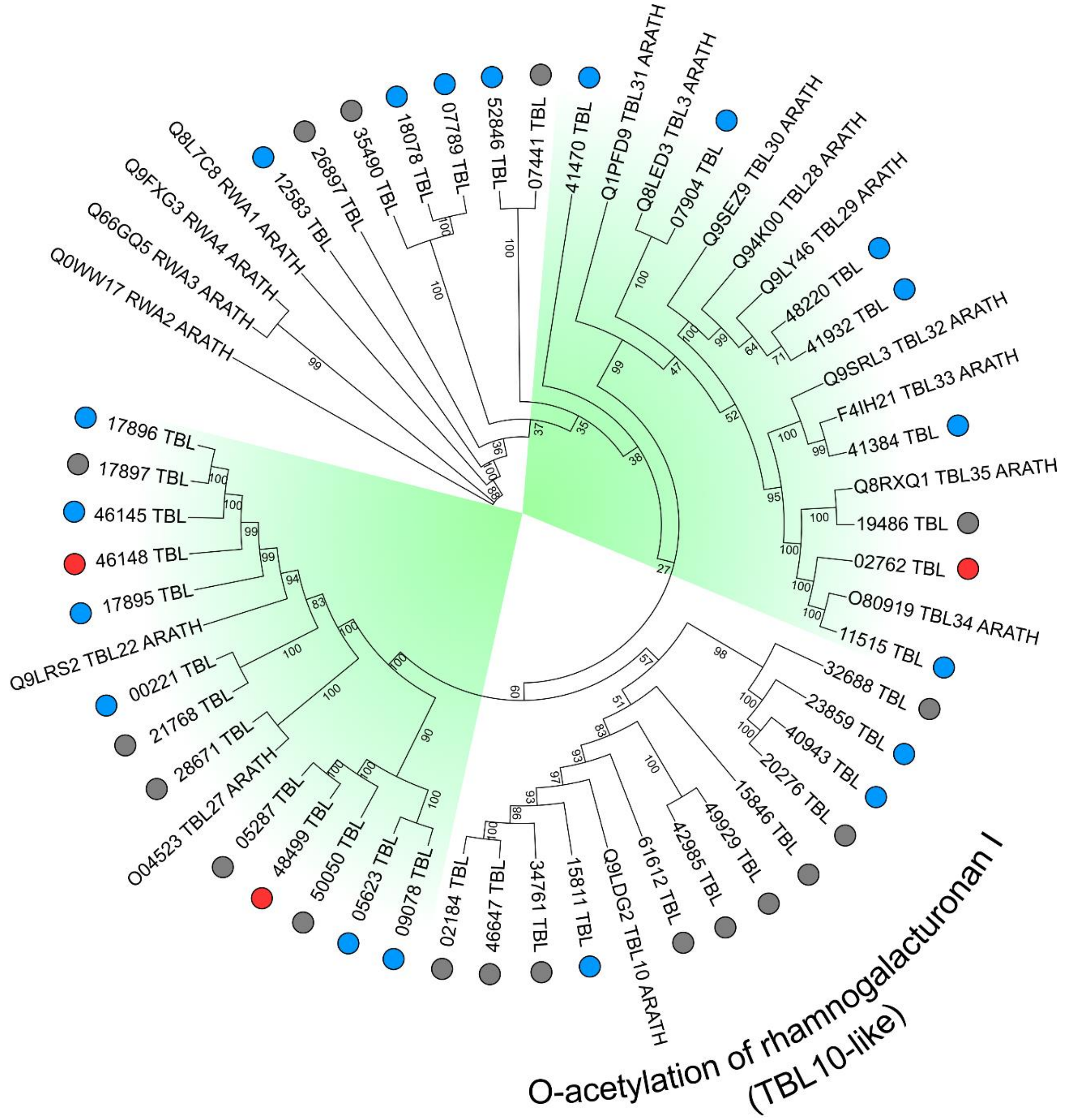

Figure S3. Phylogeny of potato TRICHOME BIREFRINGENCE-LIKE (TBL) proteins and of a reference set of *Arabidopsis thaliana* TBL proteins. The clades for TBLs acting towards xylan are highlighted in green. Potato proteins are indicated with circles according to the expression pattern of corresponding genes in *Pectobacterium atrosepticum*-infected vs. non-infected potato plants: red—upregulated; blue—downregulated; grey—non-DEG; empty—not expressed in our study. The UniProt IDs of *A. thaliana* proteins and the corresponding taxa names are given in the leaf names. For potato proteins only parts of PGSC IDs are given; the "PGSC0003DMP4000" symbols, the constant part in the beginning of all presented PGSC protein IDs, were omitted for the sake of brevity.
